# Safeguarding seniors in the digital age: an experimental study on the influence of cybersecurity awareness training on technology adoption, security behaviors, and cybercrime consciousness

**DOI:** 10.3389/fpubh.2026.1780758

**Published:** 2026-05-29

**Authors:** Mohamed Hussein Ramadan Atta, Sameer A. Alkubati, Marwa Ibrahim Mahfouz Khalil, Nawara Khirallah Abd El Fatah, Rajeh Saad Alsololi, Muthbat Majad Aldawsari

**Affiliations:** 1 Nursing Department, College of Applied Medical Sciences, Prince Sattam Bin Abdulaziz University, Wadi Addawasir, Saudi Arabia; 2 Medical-Surgical Nursing, College of Nursing, University of Hail, Hail, Saudi Arabia; 3 Gerontological Nursing, Faculty of Nursing, Alexandria University, Alexandria, Egypt; 4 Gerontological Nursing, Faculty of Nursing, Damanhour University, Damanhour, Egypt; 5 Computer and Information Engineering, College of Engineering, Prince Sattam Bin Abdulaziz University, Wadi Addawasir, Saudi Arabia; 6 Applied College, Prince Sattam Bin Abdulaziz, Wadi Ad-Dawasir, Saudi Arabia

**Keywords:** cyber literacy, cybercrime awareness, cybersecurity, digital inclusion, digital safety, older adults, security behaviors, technology acceptance

## Abstract

**Introduction:**

The rapid expansion of digital engagement has also exposed older adults to an increasing spectrum of cyber threats. Many older individuals encounter distinct challenges when navigating digital environments, including limited prior exposure to technology, lower levels of cybersecurity literacy, and heightened susceptibility to online misinformation. These vulnerabilities not only compromise their digital safety but also undermine their confidence in adopting and effectively using technology.

**Objectives:**

This study aimed to examine the effect of cybersecurity awareness training on technology adoption, cybersecurity behaviors, and cybercrime awareness among older adults residing in assisted living facilities.

**Methods:**

A quasi-experimental pretest-posttest control group design was employed. A total of 160 older adults were recruited through convenience sampling. Participants were divided into intervention and control groups. The intervention group underwent a five-week cybersecurity training program, with weekly sessions of 60–90 min. Topics included password security, phishing awareness, social media safety, and secure browsing. Validated assessment tools were used to evaluate changes in cybersecurity awareness, technology acceptance, and security practices before and after the intervention.

**Results:**

Post-intervention assessments showed that the intervention group experienced significant improvements in digital safety knowledge, attitudes toward technology use, control over digital tools, and awareness of cyber threats, especially on social media. Enhanced adoption of cybersecurity behaviors in daily life was also observed.

**Conclusion:**

Cybersecurity awareness training is effective in improving older adults’ knowledge of digital threats, encouraging positive attitudes toward technology, and promoting safe online behavior. These findings underscore the potential of targeted interventions to reduce digital vulnerability and enhance inclusion among aging populations.

## Introduction

The digital age, marked by the rapid expansion of virtual environments, digital services, and machine learning, has significantly transformed communication, globalization, and the exchange of information (1). Often described as the information stage of societal development, this era has accelerated the ways in which data are collected, processed, and analyzed (2). Technological advancement brings substantial benefits; at the same time, it introduces cybersecurity risks that affect individuals and institutions alike (3).

Cybersecurity focuses on protecting digital systems, networks, and data from unauthorized access, theft, and misuse, ensuring the confidentiality, integrity, and availability of information (4). The expansion of cloud computing and Internet of Things (IoT) technologies has further increased the importance of cybersecurity across sectors (5). For older adults, this protection extends to personal devices and sensitive information that may be vulnerable to digital threats (6).

Responsible and informed technology use is a core element of digital citizenship (7). Nevertheless, many older adults experience digital inequalities and technological stress that limit their participation in digital environments (8). Limited familiarity with online norms contributes to increased vulnerability to cybercrime (9).

### Review of literature

Evidence consistently shows that many older adults have limited awareness of common cybersecurity threats such as malware and ransomware (10). This lack of awareness contributes to digital exclusion and increases the risk of cybervictimization (11, 12). Lower levels of digital literacy further heighten susceptibility to misinformation and online scams (13).

Older adults are particularly vulnerable to social engineering and phishing attacks, partly due to higher levels of interpersonal trust (14). During the COVID-19 pandemic, many were required to adopt digital technologies without sufficient preparation, which increased their exposure to online threats (15). In addition, life transitions such as retirement may influence patterns of technology use and related security behaviors (16).

Common threats affecting this population include phishing, financial and romantic scams, impersonation fraud, ransomware, identity theft, and exposure to misinformation (17, 18). These risks are shaped by multiple factors, including digital literacy, cognitive changes, psychological well-being, and social support systems (19). Lower digital literacy is associated with increased vulnerability, whereas targeted interventions can enhance resilience (20). Age-related cognitive decline may further increase susceptibility to scams (21), while emotional distress and social isolation can intensify risk (22). Many older adults also depend on caregivers to support safe online practices (23).

Cybercrime awareness, sometimes referred to as cybercrime consciousness, is increasingly recognized as essential as older adults rely more heavily on digital tools (24). Despite this increased engagement, many older individuals still lack a meaningful understanding of cybersecurity risks. Social isolation, declining health, and reluctance to report cybercrime due to stigma or mistrust further increase the likelihood of victimization. Cognitive impairments may also limit the effectiveness of remote cybersecurity interventions (25).

Awareness alone does not necessarily lead to safe behavior. A study in Thailand reported that 75.3% of older adults had experienced fraud, particularly in rural areas, with notable emotional and social consequences (26). Similarly, research conducted in China found that seniors who frequently use mobile applications and social media, especially those managing chronic conditions, are more vulnerable to health-related online fraud (27).

Adult training programs play a critical role in strengthening digital literacy and cybersecurity skills among older populations (28). Effective programs are typically adaptable and personalized to accommodate different learning styles and levels of digital confidence (29). Practical relevance is essential, as cybersecurity concepts are more effective when linked to everyday activities such as online banking, email use, and social media engagement (30).

Hands-on exercises and real-world simulations have been shown to improve skill application and user confidence (31, 32). These approaches support safer technology use and help reduce susceptibility to scams. At the same time, several barriers remain. Resistance to change and optimism bias, where individuals underestimate their likelihood of being targeted, can limit behavioral modification (33, 34).

### Significance of the study

As older adults increasingly rely on digital technologies for communication, healthcare access, financial management, and social engagement, ensuring their cybersecurity readiness is essential. Persistent knowledge gaps and vulnerability to cyber threats necessitate targeted educational interventions.

This study addresses these needs by evaluating the effectiveness of cybersecurity awareness training designed specifically for older adults. Its findings aim to inform policymakers, educators, and community organizations about evidence-based strategies for enhancing cybersecurity literacy in this population.

Improving cybersecurity awareness among older adults contributes not only to individual protection but also to broader public safety. Digitally informed seniors are less likely to experience fraud and can promote safer online practices within their communities. Moreover, strengthening cybersecurity competence supports digital inclusion by increasing confidence and willingness to engage with technology.

Significant gaps remain in understanding the long-term effectiveness of cybersecurity training and its impact on sustained behavioral change. Few studies evaluate whether awareness translates into improved security behaviors or increased technology adoption over time. Additionally, limited research explores the relationship between cybersecurity knowledge and seniors’ willingness to engage with digital tools.

By addressing these gaps, this study contributes empirical evidence to support the development of tailored, sustainable cybersecurity interventions for older adults.

### Knowledge gap

Despite the growing body of literature on digital vulnerability among older adults, theoretical integration remains limited. While frameworks such as the Technology Acceptance Model (TAM) and its extensions are frequently cited, their application in cybersecurity contexts is often superficial, with limited examination of how perceived usefulness, ease of use, and perceived risk jointly influence protective behaviors (35, 36). Moreover, existing studies present inconsistent findings regarding the relationship between awareness and behavior; some suggest that increased exposure to digital technologies enhances competence and security practices (37), whereas others report persistent vulnerability despite frequent use, particularly among older populations (27, 38). These contradictions indicate that awareness alone may not be sufficient to produce behavioral change, highlighting a critical gap in the literature. This gap is further amplified in specific geographical contexts where digital literacy infrastructure, access to structured cybersecurity education, and public awareness initiatives are limited, yet empirical evidence from such settings remains scarce (8, 19, 39). Consequently, there is a need for theoretically grounded and context-sensitive research that moves beyond descriptive accounts. The present study addresses this gap by applying an intervention-based approach informed by TAM to examine not only changes in cybersecurity awareness but also their impact on security behaviors and technology acceptance among older adults within the studied population. In doing so, it provides a more integrated and contextually grounded understanding of digital vulnerability and resilience.

### Research hypothesis

The study hypothesizes that participation in cybersecurity awareness training will significantly improve security behaviors and technology adoption among older adults, with increased cybercrime awareness functioning as a mediating factor in this relationship.

## Materials and methods

### Study design and sample size

A quasi-experimental pretest-posttest control group study was conducted. Convenience sampling was employed to select a diverse group of older adults, representing all genders, from older adult clubs and community settings in Damanhour, Egypt. Two older adult clu bs and two community settings were chosen for the study. To prevent knowledge transfer between the groups, the control group was recruited from one older adult club and one community setting, while the intervention group, which received cybersecurity awareness education, was recruited from the remaining settings. In total, 160 participants were surveyed, with 80 assigned to the intervention group and 80 to the control group. The sample size was calculated based on an assumed effect size of 0.5 (medium effect), with an alpha error of 5%, a dropout rate of 10%, and a study power of 80%. Using G*Power software, the calculations indicated that the required total sample size was approximately 128 participants. Therefore, with an initial sample size of 160, the study had adequate power to detect a medium effect size, allowing for potential dropouts and ensuring robust statistical analysis. The development and implementation of the program occurred from January 2025 to June 2025.

### Study participants

To meet the eligibility criteria for the study, participants had to be aged 60 years or older, which was the primary focus of the research. Additionally, participants needed to possess basic computer skills, including the ability to use a computer or mobile device, navigate the internet, and send and receive emails. This foundational knowledge was essential for effectively engaging in cybersecurity training. Furthermore, all participants were required to have reliable access to the internet and a personal device, such as a computer, tablet, or smartphone, throughout the study. Willingness to participate was crucial; individuals had to be open to engaging in the cybersecurity awareness training and completing follow-up assessments to evaluate the intervention’s effectiveness. Lastly, participants needed to demonstrate sufficient cognitive ability to understand and engage with the training material, which was assessed through a brief screening tool. It was also important that participants were in stable health to ensure they could actively participate in training sessions without interruptions.

The target population of this study comprised community-dwelling older adults aged 60 years and above who actively engage with digital technologies and meet the predefined inclusion criteria. Participants were recruited using a convenience sampling approach from community centers and outpatient settings, where older adults with basic digital exposure could be accessed. The sample size was determined based on both practical feasibility and statistical considerations. Using *a priori* power analysis (e.g., G*Power), a minimum sample size was estimated to detect a moderate effect size (d = 0.5) with a significance level of 0.05 and statistical power of 0.80, which is consistent with similar intervention-based studies involving older adults. Accordingly, the final sample size was considered sufficient to identify meaningful differences between the intervention and control groups while accounting for potential attrition. Furthermore, the selected sample size aligns with previous cybersecurity and digital literacy intervention studies in older populations, which typically employ comparable sample ranges to ensure both feasibility and adequate statistical power. This approach ensures that the findings are both methodologically robust and practically applicable within the context of the study population.

## Data collection tools and methods

### Five tools were used for data collection

1- Sociodemographic, medical history, and internet-related use:

The tool is a comprehensive survey designed to gather socio-demographic, medical, and internet use data from older adults. It includes sections on socio-demographic information, such as sex, age, marital status, educational level, living arrangements, and income sufficiency. The medical data section assesses the presence and number of chronic diseases, self-reported health status, and functional independence in daily activities. Additionally, the survey explores internet use, measuring access frequency, devices used, background knowledge of internet skills, connection methods, and purposes for accessing the internet (Supplementary 1).

2- Cybersecurity awareness for cybercrime questions:

This scale, adopted from (40) research questionnaire, effectively captures various dimensions of cybercrime awareness, highlighting the importance of user knowledge in navigating the increasingly complex digital landscape. By understanding these activities, individuals can better protect themselves and contribute to a safer online community. The Cybersecurity Awareness for Cybercrime Scale includes six specific activities designed to assess users’ experiences and awareness of various cyber threats. Each item is rated on a frequency scale: “Always,” “Sometimes,” “Never,” and “Do not know.” This approach helps gauge how often individuals encounter specific cybercrime-related issues and their familiarity with these threats.

The first item addresses *phishing emails*, which are deceptive messages that often appear to be from legitimate sources, requesting sensitive information such as money, personal data, or bank account details. Phishing is a prevalent cybercrime tactic that exploits users’ trust, and awareness of such emails is crucial for preventing financial losses and identity theft.

The second item focuses on *identity theft*, a serious concern where someone steals personal information to impersonate another individual. This can involve actions such as tweeting or posting under the victim’s name, leading to reputational harm and emotional distress. Recognizing the signs of identity theft is essential for users to protect their data and respond appropriately if they become victims.

The third item examines the risk of *malware infections* on devices. Malware, including viruses and ransomware, can compromise system security, leading to data loss, unauthorized access, and significant disruptions. Awareness of malware threats is critical for users to implement protective measures, such as using antivirus software and being cautious with downloads.

The fourth item pertains to the inability to access *online services* due to cyberattacks. This could involve disruptions in banking services or other essential online functions caused by cybercriminal activities. Understanding the potential impact of cyberattacks on everyday services underscores the importance of cybersecurity awareness for maintaining access to vital resources.

The fifth item highlights the accidental encounter with *material that promotes hatred or religious extremism*. This aspect of cybercrime awareness is essential as it relates to the broader implications of online content and the responsibility of users to recognize and report harmful material. Awareness can help foster a safer online environment by encouraging proactive engagement against extremist content.

The sixth item addresses *online extortion*, where individuals may face threats demanding money to prevent harm or scandal. This form of cybercrime can lead to significant emotional and financial distress for victims. Awareness of online extortion tactics can empower users to take necessary precautions and report such incidents to authorities.

*The scoring system for the Cybersecurity Awareness for Cybercrime Scale* is designed to quantify users’ experiences and awareness of various cyber threats. Each of the six items is rated on a frequency scale, allowing respondents to indicate how often they encounter these issues with response options including “Always,” “Sometimes,” “Never,” and “Do not know.” To translate these qualitative responses into quantitative scores, a specific scoring system is applied: “Always” is assigned 3 points, “Sometimes” receives 2 points, “Never” gets 1 point, and “Do not know” is valued at 0 points. After respondents complete all six items, the total score is calculated by summing the points for each response. The cumulative score can range from a minimum of 0 (if all responses are “Do not know”) to a maximum of 18 (if all responses are “Always”). A higher total score reflects greater awareness of cybercrime risks, while a lower score indicates less awareness.

3- Short Version of Senior Technology Acceptance – 14 items:

Chen and Lou (41) introduced the Senior Technology Acceptance Model (STAM-14), a streamlined 14-item scale designed to assess technology acceptance among older adult individuals. This model is based on four key constructs: attitudinal beliefs, control beliefs, gerontechnology anxiety, and health. Participants respond using a 10-point Likert scale, where 0 indicates strong disagreement, very poor perception, high unease, and extreme dissatisfaction, while 10 reflects strong agreement, very good perception, high ease, and extreme satisfaction. The total score ranges from 14 to 140, with higher scores indicating greater technology acceptance.

Validation of the scale was carried out with 1,012 community-dwelling individuals aged 55 and older in Hong Kong, demonstrating strong internal consistency (Cronbach’s alpha = 0.817 to 0.915), construct validity (AVE = 0.455 to 0.795), and composite reliability (CR = 0.805 to 0.921). The STAM-14 exhibited robust internal reliability in this study, with Cronbach’s alpha values of 0.852 for attitudinal beliefs (3 items), 0.881 for control beliefs (4 items), 0.973 for gerontechnology anxiety (2 items), and 0.868 for health (5 items), as well as an overall STAM-14 score of 0.877.

4- Security Practices Questions:

The Security Practices Questions scale is designed to assess individuals’ behaviors and attitudes toward cybersecurity, focusing on their proactive measures to protect personal information and digital assets. This scale, adopted from (40) research questionnaire, effectively captures various aspects of security practices among users. By understanding these behaviors, individuals can enhance their cybersecurity awareness and contribute to a safer online environment.

Each question is rated on a frequency scale: “Always,” “Often,” “Sometimes,” “Seldom,” and “Never.” This approach helps to identify how frequently users engage in specific security practices, which is vital for understanding their overall cybersecurity awareness.

The first item prompts users to consider whether they check the legitimacy of a website before accessing it. This practice is crucial in preventing phishing attacks and ensuring users do not fall victim to fraudulent sites designed to steal personal information. By verifying website authenticity, individuals can significantly reduce their risk of cyber threats.

The second item addresses the tendency to create passwords that contain personal information such as last names or dates of birth. While many users may think this makes passwords easier to remember, it can weaken security. Awareness of password best practices, including using complex and unique passwords, is essential for protecting accounts from unauthorized access.

The third item raises awareness about the dangers of clicking on banners, advertisements, or pop-up screens encountered while browsing the internet. These elements can often be traps for malware or phishing attempts. Recognizing the risks associated with online advertisements is vital for maintaining cybersecurity and avoiding potential threats.

The fourth item emphasizes the importance of paying attention to privacy settings on social media accounts. Users must actively manage their privacy settings to safeguard their personal information from being accessed by unauthorized individuals or third parties. This proactive approach can help protect users from data breaches and identity theft.

The fifth item reflects a common misconception that social media services inherently protect personal information. While platforms often have security measures in place, users must remain vigilant and take additional steps to protect their data. Awareness of this reality can lead to better security practices and more informed user behavior.

The sixth item encourages individuals to read the terms and conditions carefully before using any website. Many users skip this important step, potentially overlooking critical information about data usage and privacy policies. Understanding these terms can empower users to make more informed decisions about their online activities.

The seventh item focuses on the practice of changing passwords for important accounts (such as online banking) frequently. Regularly updating passwords is a key security measure that helps mitigate the risk of account breaches. This habit can be a significant deterrent against unauthorized access.

The eighth item assesses feelings of safety when using public Wi-Fi. Many users are unaware of the vulnerabilities associated with public networks, which can expose personal data to cybercriminals. Being cautious in these environments is crucial for maintaining security and protecting sensitive information.

The ninth item addresses a common misconception that personal devices hold no value to hackers. This belief can lead to complacency in security practices. Recognizing that all devices can be targets for cyberattacks is essential for adopting a proactive approach to cybersecurity.

The tenth item highlights the importance of regularly installing software updates. Updates often contain security patches that protect against known vulnerabilities. Users who neglect this practice may leave their devices open to attacks that exploit outdated software.

Finally, the eleventh item reminds users to be careful about clicking on links in emails or social media posts. This cautious behavior can help prevent falling victim to phishing scams and other malicious activities that could compromise personal security.

5- Cybercrime Awareness on Social Media Scale:

The Cybercrime Awareness on Social Media Scale (CASM-S) is a psychometric tool developed by Arpaci and Aslan (42), aimed at measuring users’ awareness levels regarding cybercrime risks on social media platforms. The scale was created in response to the increasing prevalence of cybercrime facilitated by the vast user base and communication capabilities of social media, which have made these platforms attractive targets for cybercriminals. Understanding user awareness of the various threats they face on social media is crucial for enhancing online safety and promoting better security practices.

The CASM-S is unidimensional and consists of 22 items rated on a five-point Likert scale, with no items being reverse-coded. These items address key aspects of cybercrime awareness on social media. Examples of potential items include awareness of cyber threats, such as questions about recognizing phishing attempts or malware, understanding cyberbullying behaviors, knowledge of online scams and how to avoid them, and privacy and security practices aimed at protecting personal information. The CASM-S underwent rigorous psychometric testing to ensure its reliability and validity. The initial study involved data collection from 1,045 social media users, employing exploratory factor analysis (EFA) to identify the underlying factor structure of the scale. The results of the EFA indicated that the CASM-S possesses a unidimensional factor structure, meaning it effectively measures a single cohesive construct of cybercrime awareness. This validation process confirms that the scale is a reliable and valid instrument for assessing users’ awareness of cybercrime risks on social media.

The exploratory factor analysis conducted during the development of the CASM-S confirmed its unidimensional factor structure, indicating that all items collectively measure the overall level of cybercrime awareness among users on social media. This cohesive measurement approach enhances the scale’s effectiveness in capturing the nuances of user awareness. Although the specific scoring system for the CASM-S is not detailed in the available abstracts, it is likely to follow common psychometric practices. The items probably utilize a Likert-type scale (e.g., 1 = Strongly Disagree to 5 = Strongly Agree) to gauge the level of agreement with each statement. The total score is calculated by summing the scores of all items, with a higher total score indicating a greater level of cybercrime awareness on social media, while a lower score suggests lower awareness. Total scores can range from 22 to 110, reflecting the overall awareness of cybercrime risks.

### Ethical considerations

The ethical aspects of this research are crucial for protecting the rights and well-being of participants. We will obtain informed consent from all individuals involved, ensuring they fully understand the study’s aims, methods, potential risks, and benefits before participating. Confidentiality will be upheld, with all personal information anonymized to safeguard participants’ identities. They will also be made aware of their right to withdraw from the study at any time without affecting their medical care. Furthermore, The study protocol received approval from the Research Ethics Committee of the Faculty of Nursing, Damanhour University (RES: 111-e)on 11/4/2025. These steps are designed to create a respectful and secure research environment, fostering trust and transparency throughout the study.

## Program description and implementation

### Program overview

The program aimed to enhance cybersecurity awareness among participants, particularly seniors, by providing them with essential knowledge and practical skills to navigate the digital landscape safely. Each session focused on different aspects of information security, addressing common threats and protective measures.

### Assessment

The assessment phase began with a baseline evaluation to determine participants’ existing knowledge and attitudes toward cybersecurity. This evaluation included surveys and discussions to gauge their awareness of potential online threats.

### Planning

During the planning phase, the program developed a structured outline that defined the objectives, content, and methodologies for each session. This ensured a comprehensive approach to teaching participants about digital safety, tailored to their specific needs. The program was developed by an experienced author in cybersecurity, and the content was thoroughly reviewed and revised by a cybersecurity expert to ensure accuracy, relevance, and up-to-date best practices.

### Intervention design

1 Description of the Program Sessions:

- Frequency: Weekly sessions were held over five consecutive weeks.
- Duration: Each session lasted approximately 60 to 90 min, allowing sufficient time for content delivery and participant interaction.
- Mode: Sessions were conducted either in-person or virtually, depending on the health and availability of participants.

2 Content of the Sessions:

- Session 1: Focused on the fundamentals of information security and cybersecurity, emphasizing the risks seniors face online and strategies for protection.
- Session 2: Covered common digital threats, such as phishing and identity theft, highlighting why seniors are often targeted and how to recognize these threats.
- Session 3: Educated participants on protecting their mobile devices from hacking, discussing practical measures and signs of potential breaches.
- Session 4: Addressed malicious links and messages, teaching participants how to identify these threats and the steps to take if encountered.
- Session 5: Concentrated on the importance of strong passwords, providing tips for creating and managing passwords effectively.

3 Facilitators: The sessions were led by experienced instructors specializing in cybersecurity and digital literacy, ensuring participants received expert guidance.

### Data collection

1. Baseline Assessment: This involved gathering initial data on participants’ knowledge and experience with online security issues. Surveys were conducted to assess their familiarity with common threats and protective measures.
2. Post-Intervention Assessment: After the program concluded, a follow-up assessment was conducted to evaluate the participants’ knowledge gains and changes in behavior regarding online safety. This included surveys and discussions to measure their confidence in managing cybersecurity risks.

### Data analysis

Statistical methods: The program utilized descriptive statistics to analyze pre- and post-assessment data, identifying changes in participants’ knowledge and attitudes toward cybersecurity.

### Implementation

The implementation phase focused on delivering the planned sessions effectively. Each session followed a structured format, beginning with a presentation of key concepts, followed by interactive discussions and practical exercises. The content was designed to be engaging and relevant to the participants’ experiences. To accommodate their diverse needs, the program offered both in-person and virtual delivery options. This flexibility was particularly important in ensuring accessibility for all participants. By providing multiple avenues for engagement, the program fostered a supportive learning environment where seniors could confidently enhance their digital security skills.

## Session breakdown

### Session 1: protecting yourself in the digital world

Aim: The primary aim of this session was to educate participants about the fundamentals of information security and cybersecurity, with a specific emphasis on the importance of these topics for seniors. By the end of the session, attendees had a better understanding of how to protect themselves in the digital landscape.

Outline: The session began with an introduction to information security and cybersecurity, defining key concepts and principles, including the CIA model—confidentiality, integrity, and availability. It then highlighted the differences between information security, which encompasses all forms of information, and cybersecurity, which focuses specifically on digital threats. Following this, the session delved into the importance of cybersecurity for seniors, discussing how they could protect their personal information, avoid online fraud, and use the internet safely. The discussion also addressed the various threats and risks seniors faced, particularly phishing attacks, and explained why this demographic was frequently targeted. The session concluded with a recap of key points and an interactive discussion where participants shared their experiences with cyberattacks.

Duration: This session lasted 60 min, allowing sufficient time for both the presentation of information and audience engagement.

Audiovisual Aids: To enhance the learning experience, the session utilized several audiovisual aids. These included PowerPoint presentation slides to outline key concepts, infographics illustrating the CIA model, and short video clips showcasing phishing scenarios. Additionally, handouts summarizing the main points discussed were provided to participants for future reference.

Content: The content of the session covered the definitions and principles of information security and cybersecurity in detail. Real-life examples of cyber threats were shared to illustrate the potential dangers, particularly to seniors. The session also presented statistics on phishing attacks targeting older adults and discussed practical tips for safeguarding personal information while online. This comprehensive approach aimed to equip participants with the knowledge they needed to navigate the digital world securely.

Session Description: This session aimed to empower participants, especially seniors, with essential knowledge about information security and cybersecurity. It covered critical concepts, explained the risks they faced online, and provided practical strategies to protect personal information. The interactive nature of the session encouraged attendees to engage in discussions and share their experiences with cyber threats. By the end of the session, participants had a clearer understanding of how to safely navigate the digital landscape, enabling them to use technology with confidence.

### Session 2: common threats and how to protect yourself

Aim: This session aimed to educate participants about the common threats and risks that existed in the digital landscape, particularly focusing on how seniors could protect themselves from these dangers. By the end of the session, participants were better equipped to identify threats and take proactive measures to safeguard their personal information.

Outline: The session began with an exploration of why scammers specifically target seniors, discussing factors such as a lack of awareness regarding modern tactics, emotional manipulation, and fear-based tactics. Next, the session covered the most common threats, including phishing, viruses and malware, and identity theft. Each of these threats was defined and explained in detail. The session specifically delved into phishing, outlining its different types, providing real examples of phishing emails, and discussing how to recognize and avoid such scams. Additionally, participants learned about viruses and malware, how they spread, and methods to protect against them. The session concluded with a discussion on identity theft, explaining how personal information could be exploited and providing strategies for prevention.

Duration: This session was designed to last 60 min, allowing ample time for both the presentation of critical information and interactive discussion among participants.

Audiovisual Aids: To enhance engagement and understanding, the session utilized various audiovisual aids. These included PowerPoint slides that outlined key topics, infographics illustrating the types of phishing attacks, and examples of phishing emails. Handouts summarizing the main points and protection strategies were also provided for participants to reference after the session.

Content: The content comprehensively covered the reasons seniors were targeted by scammers, the nature of common threats such as phishing, viruses, and identity theft, and effective measures for protection. Participants learned about the different forms of phishing, including email phishing, smishing, and vishing, along with real-life examples to illustrate the tactics used by scammers. The session also addressed the dangers posed by viruses and malware and emphasized the importance of maintaining updated security measures. Finally, participants received practical advice on how to safeguard their identities online.

Session Description: This session aimed to empower participants, especially seniors, with essential knowledge about the common cyber threats they face and the protective measures they could adopt. By explaining why seniors were often targeted by scammers and detailing prevalent threats like phishing and identity theft, the session provided valuable insights into navigating the digital world safely. Participants engaged in discussions about their experiences and concerns, fostering a supportive environment where they learned from one another. Ultimately, by the end of the session, attendees felt more confident in their ability to protect themselves from online dangers.

## Session breakdown

### Session 3: protecting my phone from hacking

Aim: The primary aim of this session was to educate participants on how to protect their phones from hacking. It focused on practical steps to enhance mobile security and recognize signs of potential breaches, ensuring participants felt empowered to safeguard their devices.

Outline: The session began with a discussion on how to protect phones from hacking, outlining essential steps such as using strong passwords, avoiding untrusted apps, and keeping devices updated. Participants learned about the signs indicating that their phones might have been compromised, including unusual slowness and excessive battery drain. The discussion then shifted to actions to take if a phone was suspected to be hacked, including disconnecting from the internet and deleting suspicious apps. The session also covered the importance of using security software and strategies to avoid phone scams. Finally, participants explored the concept of two-factor authentication, its significance, and how to implement it effectively.

Duration: This session lasted 60 min, providing sufficient time for both the presentation of critical information and interactive discussions among participants.

Audiovisual Aids: To enhance engagement and understanding, the session utilized various audiovisual aids. These included PowerPoint slides that outlined key topics, infographics illustrating security steps, and examples of suspicious activity. Handouts summarizing the main points and protective measures were also provided for participants to reference after the session.

Content: The content comprehensively covered the steps to protect phones from hacking, the signs of potential breaches, and the appropriate actions to take if a device is compromised. Participants learned about the importance of antivirus software and effective strategies to avoid scams. The session also emphasized the significance of two-factor authentication and provided practical advice on how to secure social media accounts. This thorough approach aimed to equip participants with the knowledge they needed to use their devices safely.

Session Description: This session aimed to empower participants with essential knowledge about protecting their phones from hacking. It provided practical strategies for enhancing mobile security and recognizing signs of compromise. By discussing the importance of security software and two-factor authentication, the session fostered a supportive environment for participants to engage in discussions about their experiences. Ultimately, attendees left feeling more confident in their ability to secure their devices and protect their personal information.

## Session breakdown

### Session 4: malicious links and messages

Aim: The primary aim of this session was to educate participants about malicious links and messages that can threaten their online security. By the end of the session, attendees gained a better understanding of how to identify and protect themselves from these dangers.

Outline: The session began with a clear definition of malicious links and messages, explaining what they are and their potential risks. It then covered various types of threats, including phishing, fake links, malware attachments, extortion emails, and more. Participants learned how to recognize suspicious messages and links by identifying common warning signs. The session also provided practical advice on protective measures and outlined steps to take if participants encountered suspicious content. Finally, the session included interactive exercises where participants analyzed examples and classified links.

Duration: This session lasted 60 min, allowing ample time for both an informative presentation and interactive engagement with the participants.

Audiovisual Aids: To enhance understanding and engagement, the session utilized several audiovisual aids. These included PowerPoint slides outlining key concepts, infographics detailing types of malicious links and messages, and real examples for analysis. Handouts summarizing the main points and protective strategies were also provided for future reference.

Content: The content of the session comprehensively covered definitions of malicious links and messages, types of threats, and warning signs to look for. Participants learned practical strategies to protect themselves, such as verifying sources, using antivirus software, and recognizing fake communications. The session also included detailed steps for responding to suspicious messages and links, reinforcing the importance of cybersecurity awareness.

Session Description: This session aimed to empower participants with essential knowledge about recognizing and countering malicious links and messages. It provided critical information on the nature of these threats, practical strategies for protection, and interactive exercises to reinforce learning. By the end of the session, attendees felt more confident in their ability to navigate the digital landscape securely and protect their personal information from potential threats.

### Session 5: passwords–importance and tips for creating them

Aim: This session aimed to educate participants on the significance of strong passwords and provide practical tips for creating and managing them effectively.

Outline: The session covered the definition of passwords, their importance in protecting personal information, the consequences of weak passwords, characteristics of strong passwords, common mistakes to avoid, methods for securely storing passwords, and steps to take if passwords are forgotten. It also included practical exercises for creating strong passwords.

Duration: The session lasted for approximately 90 min, allowing ample time for discussion, exercises, and Q&A.

Audiovisual Aids: The session utilized a PowerPoint presentation to highlight key points, along with handouts summarizing the main concepts and examples of strong and weak passwords.

Content: The content included an introduction to the concept of passwords, a discussion on why they are important, and an outline of the characteristics that make a password strong. Participants learned about common mistakes to avoid when creating passwords and strategies for securely storing them. The session also featured practical exercises that encouraged participants to create and improve their passwords.

### Session description

The session emphasized the critical role of passwords in safeguarding personal and financial information online. Participants explored the various aspects of password security, including the necessity for complexity and regular updates. They engaged in practical exercises that demonstrated how to transform weak passwords into strong ones, reinforcing the importance of good security practices. Overall, the session aimed to empower participants with the knowledge and tools necessary to enhance their digital security through effective password management.

### Program evaluation

Immediately following the implementation of the program, the evaluation phase assessed the effectiveness of the cybersecurity training intervention. Researchers compared post-intervention knowledge and confidence scores with the baseline data collected at the beginning of the sessions. To achieve this, the same structured questionnaires were administered to participants right after the program concluded. This method enabled a thorough analysis of the intervention’s impact on participants’ cybersecurity awareness and behaviors.

## Results

### Statistical analysis

Data was processed and analyzed using IBM SPSS software version 23.0. Qualitative data were summarized as numbers and percentages, while quantitative data were characterized by means and standard deviations. The analysis included several tests: Cronbach’s Alpha was employed to evaluate reliability; the Chi-square test was used for categorical variables to compare different groups; Monte Carlo correction was applied for the Chi-square test when over 20% of cells had expected counts below 5; the Student *t*-test was utilized for normally distributed quantitative variables to compare two groups; the paired *t*-test was used for normally distributed quantitative variables to compare two time periods; and Partial Eta Squared (η^2^) was calculated to determine the effect size of the program, categorized as small, medium, or large. Statistical significance was set at *p* ≤ 0.05.

To enhance inferential rigor, additional analytical considerations were incorporated. Baseline equivalence between the intervention and control groups across socio-demographic, medical, and internet-use variables (*p* > 0.05) suggests minimal confounding influence and supports internal validity; however, potential residual confounding was acknowledged given the quasi-experimental design. Effect sizes were systematically interpreted using Partial Eta Squared (η^2^), which demonstrated large intervention effects across key outcomes, including cybersecurity awareness (η^2^ = 0.616), technology acceptance dimensions (η^2^ ranging from 0.935 to 0.980), and security practices (η^2^ = 0.761), indicating that observed differences were not only statistically significant but also practically meaningful. In addition, bias considerations were addressed. Convenience sampling may introduce selection bias, as participants with prior digital engagement were more likely to enroll, potentially limiting generalizability. Measurement bias was minimized through the use of standardized tools with established reliability (Cronbach’s Alpha), although self-reported responses may still be subject to social desirability effects. Furthermore, attrition bias was controlled by excluding participants who did not complete all study phases, ensuring consistency in pre–post comparisons.

Table 1 presents a comprehensive comparison of socio-demographic and medical characteristics between the two groups: an intervention group (*n* = 80) and a control group (*n* = 80). The data reveal no statistically significant differences between the two groups across various variables, as indicated by the chi-square (*χ*^2^) and Monte Carlo (MCp) tests.

**Table 1:** Socio-demographic and medical characteristics (*N* = 160).

Variable	Intervention (*n* = 80) *n* (%)	Control (*n* = 80) *n* (%)	*p*-value
Sex			0.631
Female	45 (56.3)	48 (60.0)	
Male	35 (43.8)	32 (40.0)	
Age groups			0.328
60–<75	38 (47.5)	46 (57.5)	
75–<85	26 (32.5)	24 (30.0)	
≥85	16 (20.0)	10 (12.5)	
Mean ± SD	73.33 ± 9.08	72.59 ± 8.50	0.596
Marital status			0.325
Married	51 (63.8)	42 (52.5)	
Others	29 (36.2)	38 (47.5)	
Education			0.638
≤Secondary	35 (43.7)	30 (37.5)	
University+	45 (56.3)	50 (62.5)	
Income			0.747
Not enough	47 (58.8)	49 (61.3)	
Enough	33 (41.3)	31 (38.8)	
Chronic disease			0.633
Yes	46 (57.5)	43 (53.8)	
No	34 (42.5)	37 (46.3)	
Functional status			0.423
Independent	62 (77.5)	67 (83.8)	
Others	18 (22.5)	13 (16.2)	

The gender distribution is relatively balanced, with 56.3% of the intervention group being female compared to 60.0% in the control group. The *χ*^2^ value of 0.231 and a *p*-value of 0.631 suggest no significant difference in sex distribution between the two groups. The age distribution also shows no significant difference, with the majority of participants in both groups aged 60 to 75 years. The mean age for both groups is similar, with a t-value of 0.531 (*p* = 0.596).

The majority of participants in both groups are married (63.8% in the intervention group and 52.5% in the control group). The absence of significant differences is indicated by the *χ*^2^ value of 3.471 (*p* = 0.325). A significant portion of participants in both groups have university education or higher, comprising 56.3% in the intervention group and 62.5% in the control group. The *χ*^2^ value of 1.796 (*p* = 0.638) indicates no statistically significant difference in education levels. The distribution of occupations before retirement shows some variability, particularly in professional and academic work, which is more prevalent in the control group (40.0%). However, the differences are not statistically significant (*χ*^2^ = 5.008, *p* = 0.171).

The majority of participants in both groups reported inadequate monthly income (58.8% in the intervention group vs. 61.3% in the control group), with no significant differences (*χ*^2^ = 0.104, *p* = 0.747). Most participants live with family, with no significant differences observed (*χ*^2^ = 2.238, *p* = 0.327). A majority of participants in both groups have chronic diseases, with 57.5% in the intervention group and 53.8% in the control group. Again, no significant differences were found (*χ*^2^ = 0.228, *p* = 0.633).

The distribution of the number of chronic diseases shows that 60.9% of those in the intervention group have no diseases compared to 76.7% in the control group. While the differences are notable, they are not statistically significant (MCp). The self-reported health status indicates that 48.8% of the intervention group rated their health as fair compared to 58.8% in the control group, with no significant difference (*χ*^2^ = 1.760, *p* = 0.415). Most participants report being independent in daily activities, though there is a slight difference in the dependent and semi-dependent categories between groups. However, the differences are not statistically significant (MCp).

Table 2 presents a comparison of internet use-related data between the intervention group (*n* = 80) and the control group (*n* = 80). The analysis indicates no statistically significant differences in internet usage patterns between the two groups, as demonstrated by the chi-square (*χ*^2^) and Monte Carlo (MCp) tests.

**Table 2:** Internet use characteristics (*N* = 160).

Variable	Intervention *n* (%)	Control *n* (%)	*p*-value
Frequency of use			0.789
Daily/almost daily	41 (51.3)	38 (47.5)	
Less frequent	39 (48.7)	42 (52.5)	
Main device			0.764
Smartphone	50 (62.5)	47 (58.8)	
Others	30 (37.5)	33 (41.2)	
Digital skill level			0.692
Beginner	49 (61.3)	44 (55.0)	
≥Intermediate	31 (38.7)	36 (45.0)	
Connection type			0.624
Mobile/Wi-Fi	75 (93.7)	72 (90.0)	
Wired	5 (6.3)	8 (10.0)	
Main purpose			0.968
Communication	34 (42.5)	32 (40.0)	
Other uses	46 (57.5)	48 (60.0)	

Regarding the frequency of internet access in the past month, 13.8% of the intervention group reported accessing the internet once or twice, while 17.5% of the control group indicated the same. A few times a week, both groups had an equal representation of 35.0, and 51.3% of the intervention group accessed the internet every day or almost daily, compared to 47.5% in the control group. The *χ*^2^ value of 0.474 (*p* = 0.789) suggests no significant difference in frequency of use.

When examining the devices used to access the internet, smartphones were the most common choice, with 62.5% of the intervention group using them compared to 58.8% of the control group. Other devices included computers (6.3% vs. 10.0%), laptops (22.5% vs. 20.0%), and tablets (8.8% vs. 11.3%). The *χ*^2^ value of 1.153 (*p* = 0.764) indicates no significant differences in device preferences.

Participants were also asked to assess their background knowledge of internet use. Among the intervention group, 61.3% classified themselves as beginners compared to 55.0% in the control group. Intermediate users comprised 36.3% of the intervention group and 41.3% of the control group, while expert users represented 2.5 and 3.8%, respectively. The MCp results (0.796, *p* = 0.692) suggest no significant differences in self-assessed internet knowledge.

In terms of connection methods, 32.5% of the intervention group used private Wi-Fi, while 31.3% of the control group did the same. Public Wi-Fi was used by 23.8% of the intervention group and 28.8% of the control group. Mobile cellular connections were reported by 37.5% of the intervention group compared to 30.0% in the control group, with a *χ*^2^ value of 1.760 (*p* = 0.624) indicating no significant differences. Wired broadband users constituted 6.3% in the intervention group and 10.0% in the control group.

Participants were also asked about their reasons for accessing the internet. The main reason across both groups was for communication and social networking, with 42.5% in the intervention group and 40.0% in the control group. Other reasons included obtaining online services (5.0% vs. 6.3%), governmental services (6.3% vs. 8.8%), educational purposes (8.8% vs. 11.3%), local and worldwide news (30.0% vs. 27.5%), and ways to volunteer or help in the community (7.5% vs. 6.3%). The *χ*^2^ value for educational purposes was 0.933 (*p* = 0.968), indicating no significant differences.

Table 3 summarizes the results of a study assessing cybersecurity awareness for cybercrime among older adults in two groups: an intervention group (*n* = 80) and a control group (*n* = 80). The data presented includes pre-test and post-test scores, highlighting significant differences in cybersecurity awareness between the groups.

**Table 3:** Cybersecurity awareness scores.

Measure	Intervention Mean ± SD	Control Mean ± SD	*p*-value	Effect size (η^2^)
Pre-test	11.41 ± 3.20	11.55 ± 2.64	0.768	—
Post-test	16.14 ± 1.86	11.79 ± 2.88	<0.001*	0.616
Within-group change	<0.001*	0.074	—	—

*p < 0.001.

In the pre-test phase, the total score for the intervention group was 11.41 ± 3.20, while the control group scored slightly higher at 11.55 ± 2.64. The *t*-test results (t1 = 0.296, p1 = 0.768) indicate no significant difference in pre-test scores between the two groups, suggesting that both groups began with a comparable level of cybersecurity awareness.

In the post-test assessment, the intervention group demonstrated a substantial increase in their total score, achieving 16.14 ± 1.86. In contrast, the control group only reached a total score of 11.79 ± 2.88. The *t*-test results reveal a statistically significant difference between the two groups (t1 = 11.359, *p* < 0.001), indicating that the intervention significantly improved cybersecurity awareness among participants.

The mean percent score further elucidates these findings. In the post-test, the intervention group had a mean percent score of 42.24 ± 7.75, while the control group scored 24.11 ± 11.98. The paired sample *t*-test results (P2 < 0.001 for the intervention group) confirm that the intervention was effective in enhancing awareness, whereas the control group showed no statistically significant change (P2 = 0.074).

The partial eta squared (η^2^) values provide additional insight into the effect size of the intervention. The η^2^ value of 0.616 for the intervention group suggests a large effect, indicating that the intervention had a substantial impact on improving cybersecurity awareness. Conversely, the η^2^ value of 0.040 for the control group indicates a negligible effect. Table 4 presents the results of a study assessing technology acceptance among older adults, comparing an intervention group (*n* = 80) and a control group (*n* = 80). The data includes pre-test and post-test scores across various dimensions of technology acceptance, highlighting significant differences attributable to the intervention.

**Table 4:** Technology acceptance outcomes.

Domain	Intervention post Mean ± SD	Control post Mean ± SD	*p*-value	Effect size
Attitudinal beliefs	27.19 ± 1.75	7.53 ± 2.98	<0.001*	0.980
Control beliefs	23.34 ± 3.76	6.51 ± 2.22	<0.001*	0.946
Anxiety	3.60 ± 1.23	18.16 ± 1.72	<0.001*	0.980
Health conditions	24.24 ± 5.36	23.76 ± 4.82	0.475	0.015
Overall score	78.36 ± 6.45	56.10 ± 6.77	<0.001*	0.935

*p < 0.001.

In the pre-test phase, both groups demonstrated similar attitudinal beliefs, with the intervention group scoring 7.71 ± 2.31 and the control group scoring 7.93 ± 2.54. The *t*-test results (t1 = 0.554, p1 = 0.580) indicate no significant difference in pre-test scores. However, in the post-test, the intervention group achieved a total score of 27.19 ± 1.75, while the control group scored only 7.53 ± 2.98. The significant difference (t1 = 50.926, *p* < 0.001) highlights the effectiveness of the intervention in enhancing attitudinal beliefs regarding technology. The mean percent scores in the post-test further illustrate this improvement, with the intervention group scoring 89.58 ± 6.48 compared to 16.76 ± 11.02 in the control group.

For control beliefs, the pre-test scores were also comparable: 6.25 ± 1.25 for the intervention group and 6.13 ± 1.23 for the control group, with no significant difference (t1 = 0.639, p1 = 0.524). In the post-test, however, the intervention group scored 23.34 ± 3.76, while the control group only reached 6.51 ± 2.22, indicating a significant improvement (t1 = 34.468, *p* < 0.001). The mean percent scores reflect a similar trend, with the intervention group at 53.72 ± 10.45, compared to 6.98 ± 6.16 for the control group.

When examining gerontechnology anxiety, both groups had similar pre-test scores (18.26 ± 1.35 for the intervention and 18.38 ± 1.46 for the control), resulting in no significant difference (t1 = 0.506, p1 = 0.613). However, the post-test scores show a dramatic change, with the intervention group scoring 3.60 ± 1.23 and the control group at 18.16 ± 1.72. The significant difference (t1 = 61.523, *p* < 0.001) indicates that the intervention effectively reduced anxiety related to technology use.

In terms of health conditions, both groups had comparable pre-test scores (23.81 ± 5.49 for intervention and 23.76 ± 4.82 for control), with no significant difference (t1 = 0.061, p1 = 0.951). Post-test scores remained similar, with the intervention group at 24.24 ± 5.36 and the control group at 23.76 ± 4.82, resulting in no significant changes (*p* = 0.475).

The overall tool assessment reflects similar trends. Pre-test total scores were nearly identical (56.04 ± 6.29 for intervention and 56.19 ± 5.76 for control), with no significant difference (t1 = 0.157, p1 = 0.724). However, post-test results indicate a significant improvement in the intervention group (78.36 ± 6.45) compared to the control group (56.10 ± 6.77), with a t-value of 21.288 (*p* < 0.001). The mean percent scores also showed a substantial increase for the intervention group in the post-test (48.73 ± 4.20) versus the control group (38.78 ± 4.45).

Table 5 presents the findings from a study evaluating security practices and cybercrime awareness among older adults, comparing an intervention group (*n* = 80) and a control group (*n* = 80). The results indicate significant improvements in both security practices and cybercrime awareness following the intervention.

**Table 5:** Security practices and cybercrime awareness.

Outcome	Intervention post Mean ± SD	Control post Mean ± SD	*p*-value	Effect size
Security practices	27.78 ± 2.72	17.96 ± 4.16	<0.001*	0.761
Social media awareness	95.35 ± 8.54	31.01 ± 7.52	<0.001*	0.976

Effect size	η^2^
Small effect	<0.5
Medium effect	0.5 ≤ 0.8
Large effect	>0.8

*p < 0.001.

In the pre-test phase for security practices, the intervention group scored 18.35 ± 4.28, while the control group scored slightly lower at 18.13 ± 4.07. The *t*-test results (t1 = 0.341, p1 = 0.734) show no significant difference in pre-test scores, indicating that both groups had a comparable baseline level of security practices. However, in the post-test, the intervention group exhibited a notable increase in their total score, achieving 27.78 ± 2.72, compared to the control group’s score of 17.96 ± 4.16. The significant difference (t1 = 17.662, *p* < 0.001) underscores the effectiveness of the intervention in enhancing security practices. The mean percent scores further highlight this improvement, with the intervention group scoring 63.13 ± 6.19, while the control group only reached 40.82 ± 9.45. The effect size (η^2^ = 0.761) indicates a large effect, suggesting that the intervention had a substantial impact on improving security practices among participants.

For cybercrime awareness on social media, pre-test scores were similar, with the intervention group scoring 30.98 ± 4.56 and the control group scoring 32.04 ± 7.61, resulting in no significant difference (t1 = 1.071, p1 = 0.286). However, the post-test results reveal a dramatic increase in scores for the intervention group, which achieved 95.35 ± 8.54, while the control group only scored 31.01 ± 7.52. The significant difference (t1 = 50.566, *p* < 0.001) indicates that the intervention effectively enhanced cybercrime awareness among older adults. The mean percent scores also illustrate this improvement, with the intervention group scoring 83.35 ± 9.70 compared to the control group’s 10.24 ± 8.55. The effect size for this dimension (η^2^ = 0.976) further confirms a large effect, demonstrating the intervention’s profound impact on participants’ awareness of cybercrime related to social media.

### Reliability of the scales

Reliability Statistics

**Table:** 

Scales	Cronbach’s Alpha
Tool 2: Cybersecurity awareness for cybercrime questions	0.883
Tool 3: Short Version of Senior Technology Acceptance	0.901
Tool 4: Security Practices Questions	0.876
Tool 5: Cybercrime Awareness on Social Media Scale	0.829

## Discussion

The present study contributes to the growing literature on digital vulnerability among older adults by demonstrating that structured cybersecurity training leads to significant improvements in awareness, security behaviors, and technology acceptance. While prior studies have consistently reported that older adults are at increased risk due to lower digital literacy, cognitive decline, and higher interpersonal trust (6, 43), most have relied on descriptive or correlational designs. In contrast, the current findings provide empirical support for the causal impact of targeted educational interventions. This aligns with experimental evidence reported by Havers et al. (44) and Chen et al. (27), yet extends their conclusions by showing that improvements are not limited to awareness but also translate into measurable behavioral change and attitudinal shifts.

Baseline equivalence between the intervention and control groups—particularly regarding internet use frequency, preferred devices, and perceived digital competence—suggests that digital engagement patterns among older adults are relatively stable. This is consistent with the Technology Acceptance Model (35), which posits that perceived usefulness and familiarity drive technology use. However, unlike studies conducted in highly digitized settings (e.g., (36)), where higher baseline digital skills are reported, the present sample reflects a context where access does not necessarily imply competence. This supports digital inequality frameworks (39), which emphasize disparities not only in access but also in the quality and safety of technology use. The findings therefore highlight a contextual gap in cybersecurity preparedness that may be more pronounced in regions where formal digital literacy programs are limited.

A key contribution of this study is its challenge to the assumption that passive exposure to technology improves cybersecurity awareness. While some literature suggests that familiarity with digital tools may lead to gradual skill acquisition (37), the current results contradict this notion. Despite similar baseline exposure, only the intervention group demonstrated significant improvements, indicating that experiential use alone is insufficient for developing protective behaviors. This finding is consistent with Havers et al. (24, 25, 38, 44), who reported persistent vulnerability among frequent internet users, but contrasts with studies suggesting incremental learning effects. The discrepancy may be explained by differences in educational context and the absence of structured training in certain geographical settings.

Importantly, the observed improvements extended beyond awareness to include enhanced technology acceptance, reduced anxiety, and increased perceived behavioral control. While previous research has linked digital training to improved competence (45, 46), few studies have examined its psychological impact. The present findings suggest that cybersecurity education may function as both a cognitive and emotional intervention, addressing fear and uncertainty associated with digital environments. This supports and extends the Technology Acceptance Model (35), indicating that perceived security may act as an additional determinant of technology adoption, particularly in older populations.

The lack of significant changes in health-related conditions further delineates the scope of the intervention. Unlike broader psychosocial interventions, the effects observed in this study appear domain-specific, targeting digital cognition and behavior rather than general well-being. This contrasts with some studies reporting secondary psychological benefits of digital inclusion programs (45), suggesting that the impact of training may depend on its content and objectives. The specificity of outcomes in the current study strengthens the internal validity and supports a more precise interpretation of intervention effects.

Finally, the strong impact of the intervention on security practices related to social media threats underscores the importance of contextual and scenario-based learning. Consistent with Sheil et al. (47), participants responded more effectively to practical, real-world examples than to abstract information. However, the magnitude of improvement observed in this study may also reflect baseline gaps in awareness within the sampled population, which could be influenced by regional differences in cybersecurity exposure and public education initiatives. This suggests that the effectiveness of similar interventions may vary across geographical contexts, highlighting the need for culturally and contextually adapted training programs.

Overall, this study advances the field by moving beyond descriptive accounts of vulnerability and providing comparative, intervention-based evidence. It demonstrates that cybersecurity training is not merely informational but plays a critical role in shaping behavior, confidence, and technology engagement. By situating these findings within both theoretical frameworks and geographical context, the study offers stronger justification for integrating structured cybersecurity education into digital inclusion strategies targeting older adults.

## Conclusion

This study demonstrates that targeted cybersecurity interventions can significantly improve both security practices and cybercrime awareness among older adults. Participants who received structured training showed notable gains in their understanding and application of online safety measures, along with heightened awareness of emerging threats, particularly those associated with social media use. These findings highlight the ongoing need for educational initiatives tailored specifically to older adults, as such programs equip them with the skills and confidence required to navigate the digital environment safely. Overall, the results provide strong evidence supporting the effectiveness of structured interventions in fostering safer online behaviors in this population.

## Recommendations

Based on the study’s findings, organizations and community programs serving older adults are encouraged to implement regular, structured educational interventions that focus on cybersecurity awareness and safe online practices. These programs should be tailored to the unique needs and challenges of older adults, offering practical, hands-on training grounded in real-life cyber threat scenarios. Interactive and engaging formats, such as workshops and small-group sessions, may enhance motivation, sustain interest, and reinforce learning.

In addition to initial training, ongoing support should be made available to older adults. This may include follow-up workshops, online learning materials, and helplines to address questions related to technology use or suspicious online activity. Collaboration among community organizations, technology providers, and law enforcement can also help establish a comprehensive support system that promotes online safety. By fostering a culture of continuous learning and support, older adults can be better equipped to navigate digital environments confidently and securely.

### Study strengths and limitations

This study has several strengths that enhance the credibility and rigor of its findings. The inclusion of both an intervention and a control group enabled a clear comparison of outcomes and strengthened internal validity by isolating the effect of the cybersecurity training. The relatively large sample size (*n* = 160) supports statistical power and enhances the potential generalizability of findings to similar older adult populations. Additionally, the use of validated assessment instruments increased the reliability and consistency of measurements related to cybersecurity awareness, technology adoption, and security behaviors. The structured training intervention, grounded in practical application, also enhances the study’s practical relevance for community and policy implementation.

Despite these strengths, several limitations should be acknowledged. First, the study hypothesis examined a relationship that may appear intuitive—namely, that cybersecurity training improves awareness and technology-related behaviors. While this relationship is theoretically established, empirical evidence specific to older adult populations remains limited. Therefore, the contribution of this study lies in providing age-specific, controlled evidence rather than introducing a novel theoretical association. Second, reliance on self-reported measures may introduce response bias, including social desirability or overestimation of behavioral change. Third, the absence of long-term follow-up limits understanding of whether observed improvements are sustained over time. Finally, participant characteristics such as prior technology exposure and baseline digital literacy may restrict broader applicability to more digitally excluded or cognitively vulnerable older populations. Future research should incorporate longitudinal designs, objective behavioral measures, and more diverse samples to strengthen evidence regarding the durability and scalability of cybersecurity training interventions.

### Clinical implications

The findings carry important implications for healthcare providers and organizations supporting older adults. The demonstrated effectiveness of targeted educational interventions underscores the value of integrating cybersecurity awareness training into routine health promotion and wellness programs. By equipping older adults with the knowledge and skills necessary to navigate the digital world safely, clinicians can help reduce stress, anxiety, and other negative health effects associated with cyber victimization.

Healthcare providers should also incorporate discussions about digital safety into routine consultations, as part of a holistic assessment of well-being. Identifying individuals at risk of cyber-related harm allows providers to recommend targeted resources or training. Partnerships between healthcare institutions, community organizations, and local agencies can facilitate broader access to cybersecurity education, promoting a safer digital environment for older adults overall.

## Future research

Future research should explore the long-term effects of cybersecurity interventions among older adults. Longitudinal studies can provide deeper insight into whether the knowledge and behaviors gained from training are retained over extended periods. Examining different delivery methods—such as in-person training, hybrid models, and online modules—may also help identify the most effective formats for diverse subgroups of older adults.

Further studies should aim to include more diverse demographic populations to improve generalizability. Research that considers cultural background, socioeconomic status, digital literacy levels, and health conditions would offer a more nuanced understanding of how various groups respond to cybersecurity training. Additionally, exploring barriers to participation—such as limited mobility, technology access, or cognitive challenges—may help inform the development of more inclusive, adaptable, and accessible interventions. Such work would contribute to creating tailored programs that support the safety and digital confidence of all older adults.

## Data Availability

The raw data supporting the conclusions of this article will be made available by the authors, without undue reservation.
