# Supplementary material for: Safeguarding seniors in the digital age: an experimental study on the influence of cybersecurity awareness training on technology adoption, security behaviors, and cybercrime consciousness
Source: Front Public Health. 2026 May 29;14:1780758. doi: 10.3389/fpubh.2026.1780758 (PMC13259838; doi:10.3389/fpubh.2026.1780758)
Supplement: Supplementary file 1 [file Data_Sheet_1.PDF]

حماية كبار السن فى العصر الرقمى: دراسة تجريبية حول تأثير التدريب على التوعية بالأمن السيبرانى فى تبنى التكنولوجيا والسلوكيات الأمنية والوعى بالجرائم السيبرانية

رقم الاستمارة:

الموافقة المستنيرة

جميع البيانات المجمعة من خلال ادوات البحث يتم حفظها في سرية تامة و لا يتم الاطلاع عليها الامن قبل الباحثين.
لديك الحق في الموافقة على المشاركة في البحث او الانسحاب في أي وقت بدون ابداء اسباب. برجاء قراءة الجمل التالية جيدا و التوقيع عليها قبل البدء بالاجابة.:

- لقد تم شرح جميع البيانات الخاصة بمشاركتي في البحث.
- لقد اتيح لي سؤال الباحثين أي اسئلة اشعر انها مهمة .
- أعلم انه يمكنني رفض المشاركة او الانسحاب في أي وقت دون ابداء اسباب.
- جميع معلوماتي الشخصية سرية و لكن قد يتم مشاركة البيانات فيما يخدم الاهداف البحثية والتعليمية كما يرى الباحثين.

اسم المشارك:	التاريخ:
رقم هاتف/واتس للتواصل:	التوقيع:

Tool 1: Sociodemographic and medical history

إستمارة البيانات الإجتماعية والديموغرافية والسريرية الخاصة بكبار السن_

أولاً :- البيانات الاجتماعية و الديموجرافية للمسن:

أولاً:- البيانات الشخصية والاجتماعية للمسن:

1. **السن :**

1- 60سنة - ( ) 2- من 75سنة- ( ) 3 - 85 سنة + ( )

2. **النوع :**

1- ذكر ( ) 2- أنثى ( )

3. **الحالة الاجتماعية:**

1- أعزب ( ) 2- متزوج ( )
3- أرمل ( ) 4- مطلق ( )

4. **المستوى التعليم:**

1- غير متعلم/ يقرا ويكتب 2- تعليم إبتدائى وإعدادى( )
3- تعليم ثانوى ( ) 4- تعليم جامعى وما فوقه ( )

5. **الدخل الشهرى:**

1- كافى( ) 2-غيركافى( )

6. **مع من تعيش:**

1- بمفردك ( ) 2- مع الأسرة( ) 3- مع الأقارب( )

ثانياً:- البيانات السريرية للمسن:

7) **هل تعانى من أمراض مزمنة:**

1- لا   2- نعم ( )

8) عدد الأمراض المزمنة:

1- مرض واحد ( )   2-من1 الى 2 ( )   3-أكثر من 2 ( )

9) ماهو تقييمك لصحتك:

1- سيئة ( )   2- متوسطة ( )   3- جيدة ( )

10) الحالة الوظيفية في الأنشطة اليومية:

1- معتمد كلياعلى غيره ( )   2- معتمد جزئيا ( )   3- مستقل تماما ( )

ثالثا:- البيانات الخاصة باستخدام الانترنت:

1. كم مرة استخدمت الإنترنت خلال الشهر الماضي؟

- كل يوم أو كل يوم تقريبًا
- عدة مرات في الأسبوع
- مرة واحدة في الأسبوع
- مرة أو مرتين
- أبدا

2. على أي الأجهزة يمكنك الوصول إلى الإنترنت؟

- الهاتف الذكي
- جهاز كمبيوتر
- كمبيوتر محمول
- كمبيوتر لوحي

3. ما هو المستوى الذي يمكن أن يمثل معرفتك الأساسية حول استخدام الإنترنت؟

- مبتدىء (الذي يمكنه الانتقال إلى صفحات ويب محددة واستخدام وسائل التواصل الاجتماعي وبعض التطبيقات)
- متوسط (لديه القدرة على تنزيل التطبيقات وإدارة إعدادات الأجهزة ولديه معرفة بالأجهزة والبرامج)
- خبير (متخصص كمبيوتر، مهندس شبكات، أو مسؤول قاعدة بيانات)

4. كيف تتصل بالإنترنت؟

- خدمة الواي فاي الخاصة (في المنزل)
- شبكة Wi-Fi عامة (على سبيل المثال في المقهى أو العمل)
- الخلوية المتنقلة
- النطاق العريض السلكي
- لا أعرف

5. لماذا تحتاج إلى الوصول إلى الإنترنت؟

- التواصل والشبكات الاجتماعية
- الحصول على الخدمات عبر الإنترنت
- الحصول على الخدمات الحكومية
- الأغراض التعليمية
- الأخبار المحلية والعالمية
- طرق التطوع أو المساعدة في المجتمع
- اخر يذكر

Tool2: cyber-security awareness questions for cybercrime

الأداة 2: أسئلة التوعية بالأمن السيبراني المتعلقة بالجرائم السيبرانية

	أسئلة التوعية بالأمن السيبراني المتعلقة بالجرائم السيبرانية	دائما	أحيانا	ابدا	لا اعرف
.1	يتم استقبال رسائل البريد الإلكتروني للتصيد الاحتيالي (على سبيل المثال، طلب المال أو المعلومات الشخصية أو تفاصيل الحساب المصرفي)				
.2	سرقة الهوية (يقوم شخص ما بسرقة بياناتك الشخصية وانتحال شخصيتك، على سبيل المثال، التغريد باسمك)				
.3	يتم إصابة الجهاز بالبرامج الضارة (مثل الفيروسات				
.4	عدم القدرة على الوصول إلى الخدمات عبر الإنترنت (مثل الخدمات المصرفية) بسبب الهجمات الإلكترونية.				
.5	التعرض عن طريق الخطأ لمواد تشجع على الكراهية أو التطرف الديني				
.6	الابتزاز عبر الإنترنت (طلب المال لتجنب أو إيقاف الابتزاز، أو لتجنب الفضيحة)				

Tool 3: Short Version of Senior Technology Acceptance – 14 items

الأداة 3: نسخة قصيرة من قبول التكنولوجيا لدى كبار السن – 14 عنصرًا

**المعتقدات الموقفية**			لا أوافق بشدة						أوافق بشدة	
**استخدام التكنولوجيا من شأنه أن يعزز فعاليتك في الأنشطة اليومية.**	1	2	3	4	5	6	7	8	9	10
**ستجد التكنولوجيا مفيدة في أنشطتك اليومية.**	1	2	3	4	5	6	7	8	9	10
**أعجبتك فكرة استخدام التكنولوجيا.**	1	2	3	4	5	6	7	8	9	10
**السيطرة على المعتقدات**			لا أوافق بشدة						أوافق بشدة	
**يمكن أن تكون ماهرًا في استخدام التكنولوجيا.**	1	2	3	4	5	6	7	8	9	10
**يمكنك إكمال مهمة ما باستخدام التكنولوجيا إذا كان هناك من يشرح لك كيفية القيام بذلك.**	1	2	3	4	5	6	7	8	9	10
**حالتك المالية لا تحد من أنشطتك في استخدام التكنولوجيا.**	1	2	3	4	5	6	7	8	9	10
**عندما تريد أو تحتاج إلى استخدام التكنولوجيا، فهي متاحة لك.**	1	2	3	4	5	6	7	8	9	10
**قلق تكنولوجيا الشيخوخة**			لا أوافق بشدة						أوافق بشدة	
**تشعر بالخوف من استخدام التكنولوجيا**	1	2	3	4	5	6	7	8	9	10
**تتردد في استخدام التكنولوجيا خوفا من ارتكاب أخطاء لا يمكنك تصحيحها**	1	2	3	4	5	6	7	8	9	10
**الظروف الصحية**			ردىء جدا						جيد جدا	
**كيف هي ظروفك الصحية العامة؟**	1	2	3	4	5	6	7	8	9	10
			غير سهل للغاية						للغاية	
**ما مدى قدرتك على التركيز؟**	1	2	3	4	5	6	7	8	9	10
			غير راض جدا						راض جدا	
**ما مدى رضاك عن علاقاتك الشخصية؟**	1	2	3	4	5	6	7	8	9	10
			غير راض جدا						راض جدا	
**ما مدى رضاك عن الدعم الذي تتلقاه من الأصدقاء والعائلة؟**	1	2	3	4	5	6	7	8	9	10
			غير راض جدا						راض جدا	
**ما مدى رضاك عن نوعية حياتك؟**	1	2	3	4	5	6	7	8	9	10

ملحوظة: تشير التكنولوجيا هنا إلى المنتجات أو الخدمات الرقمية أو الإلكترونية التي يمكن أن تزيد من الحياة المستقلة والمشاركة الاجتماعية لكبار السن بصحة جيدة نسبيًا وراحة وأمان. إذا تم استخدام هذا الإجراء لتقييم قبول تقنية معينة، فيجب تحديده بوضوح للمستخدمين المحتملين.

Tool 4 : Security Practices Questions
أداه 4:أسئلة الممارسات الأمنية

أسئلة الممارسات الأمنية						
		دائما	غالبا	احيانا	نادرا	ابدا
1.	أتحقق من شرعية موقع الويب قبل الوصول إليه					
2.	أقوم بإنشاء كلمة مرور تحتوي على معلوماتي الشخصية (مثل الاسم الأخير وتاريخ الميلاد					
3.	إنني أدرك خطورة الضغط على اللافتات أو الإعلانات أو الشاشات المنبثقة التي تظهر عند تصفح الإنترنت					
4.	أعطي الاهتمام الواجب لإعدادات الخصوصية على حساب (حسابات) وسائل التواصل الاجتماعي الخاصة بي (مثلFacebook)					
5.	خدمات وسائل التواصل الاجتماعي تحمي معلوماتي الشخصية					
6.	أقرأ الشروط والأحكام بعناية قبل استخدام أي موقع إلكتروني					
7.	أقوم بتغيير كلمات مرور الحسابات المهمة (مثل الخدمات المصرفية عبر الإنترنت) بشكل متكرر					
8.	أشعر بالأمان عند استخدام شبكة واى فاى العامة					
9.	أشعر أن أجهزتي الرقمية (الكمبيوتر والهواتف الذكية) ليس لها أي قيمة بالنسبة للمتسللين، فهم لا يستهدفونني					
10.	أقوم بتثبيت تحديثات البرامج بانتظام					
11.	أنا حريص على النقر على الروابط الموجودة في رسالة بريد إلكتروني أو منشور على وسائل التواصل الاجتماعي					

Tool 5 : Cybercrime Awareness on Social Media Scale
أداة 5: التوعية بالجرائم الإلكترونية على نطاق وسائل التواصل الاجتماعي

مقياس التوعية بالجرائم الإلكترونية على نطاق وسائل التواصل الاجتماعي						
		لا اوافق بشدة 1	لا اوافق 2	محايد 3	اوافق 4	اوافق بشدة 5
1.	يعد اختراق حساب شخص آخر على وسائل التواصل الاجتماعي دون إذن جريمة.					
2.	تعتبر مشاركة البيانات الشخصية مع أطراف ثالثة دون علم المستخدم جريمة.					
3.	هناك عقوبة لانتهاك سرية التحقيق على وسائل التواصل الاجتماعي.					
4.	يعد الترويج لاستخدام المواد الضارة على وسائل التواصل الاجتماعي جريمة.					
5.	تشكل المنشورات التي تدعم التنظيمات الإرهابية على وسائل التواصل الاجتماعي جريمة.					
6.	تشكل المنشورات السياسية والعسكرية التي لا أساس لها من الصحة على وسائل التواصل الاجتماعي جريمة.					
7.	يعد بيع منتجات غير قانونية (مزيفة أو مسروقة) على وسائل التواصل الاجتماعي جريمة					
8.	يعد توجيه الأفراد إلى مواقع غير قانونية من خلال الروابط الموجودة على وسائل التواصل الاجتماعي جريمة					
9.	تعتبر المراهنة/المقامرة غير القانونية على وسائل التواصل الاجتماعي جريمة.					
10.	"إن مشاركة الأعمال المحمية بحقوق الطبع والنشر على وسائل التواصل الاجتماعي تعتبر جريمة.					
11.	أعلم أن استخدام العبارات المسيئة على وسائل التواصل الاجتماعي يعد جريمة.					
12.	"إن تسجيل الفيديو أو الصوت على وسائل التواصل الاجتماعي دون إذن يعد جريمة.					
13.	إن إفشاء الأسرار المتعلقة بالواجبات السرية على وسائل التواصل الاجتماعي جريمة.					
14.	أعلم أن الحصول على ميزة غير عادلة من خلال المنشورات غير القانونية على وسائل التواصل الاجتماعي يعتبر جريمة.					
15.	أعلم أن مضايقة شخص آخر على وسائل التواصل الاجتماعي يعد جريمة.					
16.	أدرك أن التسلط عبر الإنترنت على وسائل التواصل الاجتماعي يعد جريمة.					
17.	"أدرك أنه لن يمكن مشاركة المحتوى الجنسي على وسائل التواصل الاجتماعي.					
18.	إن مشاركة الصور العنيفة على وسائل التواصل الاجتماعي تعتبر جريمة.					
19.	أعلم أن نشر البرامج الضارة باستخدام وسائل التواصل الاجتماعي يعتبر جريمة.					

					من الضروري تقديم شكوى جنائية ضد من ينتهك خصوصية الحياة الفردية على وسائل التواصل الاجتماعي.	**20.**
					أعلم أن الأخبار الكاذبة المنتشرة على وسائل التواصل الاجتماعي تشكل جريمة	**21.**
					تعتبر مشاركة البرامج غير المرخصة على وسائل التواصل الاجتماعي جريمة.	**22.**
